# Gender Perceptions Among Women Undergoing In Vitro Fertilization Treatment: The Role of Sociodemographic Factors

**DOI:** 10.5152/eurasianjmed.2026.261428

**Published:** 2026-06-08

**Authors:** Taha Berkay Börekçi, Bünyamin Börekçi

**Affiliations:** 1 Institute of Educational Sciences, Atatürk University, Erzurum, Türkiye; 2 Department of Gynecology and Obstetrics, Atatürk University Faculty of Medicine, Erzurum, Türkiye

**Keywords:** Gender perception, ICSI treatment, infertility, reproductive health, sociodemographic factors

## Abstract

**Background:**

Gender perception reflects how individuals interpret and internalize social norms and expectations related to femininity and masculinity. In societies where traditional gender roles remain influential, these perceptions may affect reproductive experiences and responses to infertility. However, data examining gender perception among women undergoing infertility treatment remain limited. This study aimed to examine gender perceptions among women undergoing intracytoplasmic sperm injection (ICSI) treatment and to investigate the association between gender perception and selected sociodemographic characteristics.

**Methods:**

This descriptive study was conducted at the In Vitro Fertilization and Reproductive Health Center of Atatürk University Hospital. Women aged 18-45 years undergoing ICSI treatment were included in the study using purposive sampling. Data were collected using a Demographic Information Form including variables of age, educational level, socio-economic status, upbringing culture, and the Perception of Gender Scale (PGS).

**Results:**

A total of 119 women participated in the study. Age and upbringing culture were not significantly associated with PGS scores. Educational level had a significant effect on gender perception, with participants who had a university education demonstrating more egalitarian gender perceptions compared with those with lower education levels. Socioeconomic status showed a significant overall association with PGS scores; however, post hoc comparisons did not reveal statistically significant differences between specific groups.

**Conclusion:**

Educational attainment appears to be an important factor influencing gender perception among women undergoing ICSI treatment. These findings suggest that gender-related attitudes may play a role in infertility experiences and highlight the importance of gender-sensitive approaches in reproductive health services.

**Main Points**

- Educational level was significantly associated with gender perception, with higher education linked to more egalitarian gender role attitudes.
- Age and upbringing culture did not demonstrate a clear or consistent influence on gender perception among women undergoing in vitro fertilization (IVF) treatment. Socioeconomic status has a general effect on scale scores, but the differences between groups are not clearly distinguishable.
- Understanding gender perceptions may help improve gender-sensitive approaches in infertility care and support services for women undergoing IVF treatment.

## Introduction

Gender perception refers to how individuals interpret and internalize social norms, expectations, and cultural meanings associated with femininity and masculinity.^1^ Rather than being determined solely by biological differences, gender roles are largely shaped through social, cultural, and historical processes that structure power relations between women and men.^2^^,^^3^ From a socio-cognitive perspective, gender perception develops through classification mechanisms that help individuals interpret complex social environments. Repeated exposure to culturally dominant representations leads to the formation of relatively stable cognitive schemas that guide attention, interpretation, and behavior.^4^^,^^5^ Although stereotypes function as cognitively efficient tools that reduce uncertainty, they also reinforce normative boundaries and contribute to the persistence of gendered expectations across generations.^5^^,^^6^

Gender stereotypes influence not only interpersonal interactions but also institutional structures such as education, employment, and family organization. In educational settings, gendered expectations may shape academic self-confidence, subject preferences, and career aspirations.^7^ Similarly, in the workplace, stereotypes regarding competence and leadership may influence recruitment decisions, wage distribution, and promotion opportunities.^8^ Comparative research indicates that gender roles continue to shape caregiving responsibilities across societies. For example, in the United Kingdom, men’s relatively low participation in parental leave has been associated with the persistence of traditional caregiving roles despite policy reforms promoting shared parental leave.^9^^,^^10^ Studies from France and Spain similarly demonstrate that although fathers have become more visible in childcare, most domestic and caregiving responsibilities continue to fall on women, reinforcing gender inequality.^11^^,^^12^ These findings illustrate that cultural expectations may sustain traditional divisions of labor even in contexts where formal policies promote gender equality.

In regions characterized by strong traditional social structures, gender roles may be defined more rigidly. In the Eastern Anatolia Region of Türkiye, family formation, childbearing, and parenting responsibilities are often organized within a framework of traditional gender expectations in which women are primarily associated with domestic work and childcare, while men are linked to authority and economic provision.^13^ In such contexts, motherhood is frequently perceived as a central component of female identity, and the absence of children may carry significant social implications.

Infertility therefore represents not only a medical condition but also a socially and psychologically challenging experience. Individuals facing infertility may encounter stigma, social pressure, and feelings of inadequacy, particularly in societies where fertility is strongly associated with marital stability and social legitimacy.^14^ Although in vitro fertilization (IVF) offers an important medical solution for couples experiencing infertility, the treatment process is influenced by cultural perceptions and gendered expectations.^15^^,^^16^ Previous studies in Türkiye indicate that women undergoing infertility treatment often experience substantial social pressure to fulfill the role of motherhood, which may increase stress and anxiety during treatment.^17^^,^^18^ Gender perceptions may also influence access to healthcare services, treatment adherence, and psychological adjustment during IVF treatment.^19^^,^^20^ Furthermore, infertility-related stigma has been shown to significantly affect women’s mental health and quality of life, highlighting the importance of effective social support mechanisms.^21^^,^^22^

The theoretical framework of this study is based on gender schema theory, proposed by Sandra Bem, which suggests that individuals organize gender-related knowledge and expectations into cognitive schemas that guide their perceptions and behaviors.^23^ These schemas develop through socialization processes beginning in early childhood and are reinforced through family interactions, educational environments, peer relationships, and media representations.^24^^,^^25^ Over time, they influence identity development and shape attitudes toward gender roles across different domains of life.^21^^,^^26^

Despite extensive research examining psychological distress and coping mechanisms in infertility, the concept of gender perception has received relatively limited attention in the context of infertility patients.^17^^,^^21^ Therefore, the primary aim of this study is to examine gender perceptions among individuals undergoing IVF treatment in the Eastern Anatolia Region of Türkiye and to explore how these perceptions vary according to socio-demographic and cultural factors.

## Material and Methods

This study was conducted using a descriptive research design at In Vitro Fertilization and Reproductive Health Center of Atatürk University Hospital between December 15, 2024, and December 15, 2025. Institutional permission was obtained from the Non-Interventional Clinical Research Ethics Committee of Atatürk University Faculty of Medicine with ethical approval number B.30.2.ATA.0.01.0038.

Women between the ages of 18 and 45 undergoing intracytoplasmic sperm injection at the center were eligible for the study. The sample was selected using purposive sampling.^27^^,^^28^ In purposive sampling, participants who can best answer the research questions are selected intentionally based on their relevance to the phenomenon under investigation. This approach ensures depth and contextual richness rather than statistical generalization. All eligible women were offered to participate in the study, and those who gave signed informed consent were recruited consecutively to the study.

Data were collected using the Demographic Information Form and the Perception of Gender Scale (PGS). The Demographic Information Form includes variables of age, education status, sociodemographic status, and upbringing culture. Education statuses were categorized as primary school, middle school, high school, and university; sociodemographic status as low, medium, and high; and upbringing culture as authoritarian, democratic, and liberal. For PGS, the one developed by Altınova and Duyan, consisting of 25 items rated on a 5-point Likert scale, was used.^29^ The scale has a unidimensional structure, and its Cronbach’s alpha coefficient was reported as 0.872, indicating high internal consistency.^29^ Higher scores indicate more egalitarian perceptions of gender roles.

Descriptive statistics were calculated to summarize demographic variables and scale scores. The Kolmogorov–Smirnov test was used to check the normality of distributions. Independent samples *t*-test and 1-way analysis of variance were used to examine differences between groups. Least Significant Difference (LSD) and Bonferroni tests were used for post hoc comparison for age and education level, respectively. All significance tests were 2-tailed, with a significance level of 0.05 in accordance with conventional statistical recommendations.^30^ The data were analyzed using IBM Statistical Package for the Social Sciences (SPSS) version 27.0 (IBM Corp., Armonk, NY, USA).

## Results

Demographic data and comparison of PGS scores according to the variables is demonstrated in Table 1. A total of 119 women were included in the study. Both 18-30 and 30-45 age groups are represented in the study, but the 18-30 age group has a slightly higher proportion (Table 1). The sample group relatively overrepresented women who were university graduates, belonged to the middle-income group, and whose upbringing culture was defined as democratic.

There was no statistically significant difference in PGS scores between the 18 and 30 age group and the 30 and 45 age group (*t* = −0.699, *df* = 112.397, *P* = .486).

Educational level has a significant effect on the PGS score (*F* = 12.933, *P* = .000). Participants at the university level scored significantly higher PGS scores than all other groups, and this difference is particularly noticeable compared to the middle school and elementary school groups. Furthermore, the high school group scored significantly higher than the middle school group. Perception of Gender Scale scores were similar between participants with primary and middle school education levels.

Socioeconomic status has a statistically significant effect on PGS scores (*F* = 3.091, *P* = .049). It was observed that participants with a medium socioeconomic level scored higher than the other groups. However, these differences were not found to be statistically significant in post hoc comparisons. These results indicate that socioeconomic status has a general effect on scale scores, but that differences between groups are not clearly distinguishable.

Participants who grew up in a democratic culture scored higher than the other groups, followed by those who grew up in a liberal culture and those who grew up in an authoritarian culture. However, upbringing culture does not have a significant effect on scale scores (F = 2.502, *P* = .086).

## Discussion

The findings indicate that age did not have a statistically significant effect on gender perception.^31^^,^^32^ Although developmental literature suggests that gender attitudes may evolve across the lifespan, particularly through educational exposure and social interaction, the absence of a significant age effect in this study may be explained by the relatively homogeneous age distribution of the sample and the shared sociocultural context in which participants were embedded. In regions where traditional gender norms are strongly institutionalized, age-related variation may be less pronounced, as dominant gender schemas are reinforced consistently across generations. Thus, contextual continuity may attenuate developmental differences.

In contrast, educational level emerged as a significant determinant, with higher education associated with more egalitarian gender perceptions.^33^^-^^36^ Education appears to function not merely as a source of knowledge acquisition but as a transformative socializing agent that exposes individuals to alternative normative frameworks, critical thinking skills, and diverse social environments. Increased educational attainment may facilitate questioning of traditional role prescriptions and reduce rigid adherence to stereotypical expectations. This finding is consistent with theoretical perspectives suggesting that formal education promotes reflexivity and weakens the internalization of hierarchical gender schemas.^33^^,^^34^ Moreover, institutional exposure to gender equality discourses and rights-based frameworks may further support egalitarian orientations.^35^^,^^36^

However, socioeconomic status did not produce statistically significant differences, suggesting that its effect may operate indirectly through structural and institutional mechanisms rather than through direct attitudinal shifts.^37^^,^^38^ Although socioeconomic resources can influence access to education and healthcare, the internalization of gender norms may remain stable across income levels within culturally cohesive environments. Structural inequalities may reproduce gendered expectations even when material conditions differ. In this sense, socioeconomic status may interact with institutional contexts and labor market structures rather than independently shaping gender perception.^37^^,^^38^ The lack of statistically significant group differences in this study may therefore reflect the dominance of cultural norms over purely economic variables.

Similarly, upbringing culture did not produce significant differences, although theoretical literature suggests that early family socialization plays a critical role in gender schema formation.^39^^,^^40^ One possible explanation is that family-level differences in parenting style may be overshadowed by broader community norms and regional cultural patterns. Even when families identify as democratic or liberal, they may still operate within a larger normative system that defines motherhood, caregiving, and femininity in traditional terms. Therefore, early socialization effects may not be sufficiently distinct across groups to generate measurable differences in adulthood within this context.

From a broader perspective, the findings suggest that gender perception is shaped through a complex interaction between cognitive schemas, institutional exposure, and sociocultural context. Education appears to function as a key mediating variable capable of modifying internalized schemas, whereas age, socioeconomic status, and upbringing culture may exert more subtle or indirect influences. This aligns with gender schema theory, which posits that schemas are resilient but not immutable; exposure to alternative normative systems may gradually reshape cognitive structures.^23^^,^^25^

In the specific context of IVF treatment, these findings carry important implications. More egalitarian gender perceptions may influence how women interpret infertility experiences, seek support, and engage with treatment processes. Individuals with more egalitarian orientations may be less likely to internalize infertility as a personal failure and more likely to approach treatment decisions collaboratively. Conversely, rigid gender schemas may intensify self-blame, stigma sensitivity, and emotional distress. Thus, gender perception may function as a cognitive mediator between cultural norms and psychological outcomes in infertility contexts.

Overall, education appears to be a powerful determinant in shaping egalitarian gender perceptions, whereas other variables may exert context-dependent or indirect effects. These findings highlight the importance of educational interventions and gender-sensitive health service approaches in IVF treatment contexts. Integrating gender awareness into reproductive health services may contribute to reducing stigma, enhancing psychological resilience, and improving treatment adherence. Future research should employ longitudinal and multi-level designs to examine how cognitive schemas interact with structural conditions over time and to explore potential mediating mechanisms between gender perception and health-related outcomes.

## Figures and Tables

**Table 1.** Demographic Data and Comparison of PGS Scores According to These Variables

**Variable**	**Descriptive Data (n = 119)**	**PGS Score**	*P*
Age, years			
18-30	64 (54)	91 ± 16	.486^a^
30-45	55 (46)	93 ± 16	
Education			
Primary school	13	84 ± 14	<.001^b^
Middle school	19	78 ± 10	
High school	34	89 ± 15	
University	53	100 ± 14	
Socioeconomic status			
Low	11	84 ± 13	.049^b^
Medium	92	94 ± 16	
High	16	86 ± 15	
Upbringing culture			
Authoritarian	26	86 ± 16	.086^b^
Democratic	60	95 ± 16	
Liberal	33	91 ± 15	

Data were given as number (%) or mean ± standard deviation wherever it is appropiate.

PGS, Perception of Gender Scale.

a: Independent samples t-test; b: One-way ANOVA test. Values are presented as mean ± standard deviation. Post hoc comparisons were performed using the Least Significant Difference (LSD) test where applicable.

## Data Availability

The data that support the findings of this study are available on request from the corresponding author.

## References

[1] Samuelsson M Samuelsson J. Gender differences in boys’ and girls’ perception of teaching and learning mathematics. Open Rev Educ Res. 2016;3(1):18 34. (doi: 10.1080/23265507.2015.1127770)

[2] Ito TA Urland GR. The influence of processing objectives on the perception of faces: an ERP study of race and gender perception. Cogn Affect Behav Neurosci. 2005;5(1):21 36. (doi: 10.3758/CABN.5.1.21) 15913005

[3] Kyte SB Riegle-Crumb C. Perceptions of the social relevance of science: exploring the implications for gendered patterns in expectations of majoring in STEM Fields. Soc Sci. 2017;6(1):19. (doi: 10.3390/socsci6010019) 38883188 PMC11177846

[4] Boczek K Doğruel L Schallhorn C. Gender byline bias in sports reporting. Sage Open. 2023;13(4):1 20.

[5] Morcillo JJ Clemente VJ. Gender differences in body satisfaction perception. Nutrients. 2024;16(1):10104. 10.3390/nu16010104 PMC10781077 38201935

[6] Tarhan S Ünal F Çürükvelioğlu Köksal E. Toplumsal cinsiyet kalıp yargıları ölçeği geliştirme çalışması. Pamukkale Univ Sos Bilim Enst Derg. 2024;62:353 367.

[7] Li Y Singh C. Effect of gender, self-efficacy, and interest on perception of the learning environment and outcomes in calculus-based introductory physics courses. Phys Rev Phys Educ Res. 2021;17(1):010143. (doi: 10.1103/PhysRevPhysEducRes.17.010143)

[8] Szkody E Steele EH McKinney C. Race and gender: Perception and reception of support from family and friends. Transl Issues Psychol Sci. 2021;7(4):435 450. (doi: 10.1037/tps0000251)

[9] Atkinson J. Shared Parental Leave in the UK: aan it advance gender equality by changing fathers into co-parents? Int J Law Context. 2017;13(3):356 368. (doi: 10.1017/S1744552317000209)

[10] Financial Times. Why did shared parental leave fall flat? 2024 Sep 7. Accessed 2026 February 20. https://www.ft.com.

[11] Le Monde. New Fathers, a Modern Kind of Hands-Off Dads; 2024. Accessed 2026 February 20. https://www.lemonde.fr.

[12] País E. ¿Es posible hablar del concepto “buena madre”?; 2024. Accessed 2026 February 20. https://elpais.com.

[13] Çetin F. Doğu Anadolu Bölgesi’nin sosyoekonomik yapısı üzerine inceleme. Sosyol Derg. 2018;21(3):147 165.

[14] Li Y Wang Y Song F Assessing Gender Perception Differences in Color Combinations in Digital Visual Interfaces Using Eye tracking – The Case of HUD. Int J Hum-Comput Interact. 2024;40(20):6591 6607. (doi: 10.1080/10447318.2023.2258020)

[15] Hamdani NA Ramadani V Anggadwita G Gender stereotype perception, perceived social support and self-efficacy in increasing women’s entrepreneurial intentions. Int J Entrep Behav Res. 2023;29(6):1290 1313. (doi: 10.1108/IJEBR-02-2023-0157)

[16] Greil AL Slauson-Blevins K McQuillan J. The experience of infertility: a review of recent literature. Sociol Health Illn. 2010;32(1):140 162. (doi: 10.1111/j.1467-9566.2009.01213.x) 20003036 PMC3383794

[17] Akkaş İ. Toplumsal cinsiyet algısı üzerine değerlendirme. Dünya Multidisip Araşt Derg. 2020;1:55 72.

[18] Hoşgör H Akyüz İ Cengiz E. İnfertil hastalarda tedavi bırakma faktörleri. MAKÜ Sos Bilim Enst Derg. 2017;9(19):64 84.

[19] Kahraman B Ozonsoy N Akıllı H Toplumsal cinsiyet algı araştırması. Turk Stud. 2014;9(2):811 831.

[20] Ying L Wu LH Loke AY. Gender differences in emotional reactions to in vitro fertilization treatment: a systematic review. J Assist Reprod Genet. 2016;33(2):167 179. (doi: 10.1007/s10815-015-0638-4) 26712577 PMC4759000

[21] Xie Y Zhang X Chen Y. Infertility stigma and mental health. Front Psychol. 2023;14:1093459. 10.3389/fpsyg.2022.1093459 PMC9869765 36698573

[22] Idugboe MI Umeh NJ Adewara OR Psychological impact of infertility. *Int J Res Rep Gynaecol.* 2024;7(1):88 98.

[23] Bem SL. Gender schema theory: a cognitive account of sex typing. Psychol Rev. 1981;88(4):354 364. (doi: 10.1037/0033-295X.88.4.354)

[24] Sternadori M Abitbol A. Gender roles and media effects. J Media Stud. 2022;14(2):110 125.

[25] Fan W Wang Y Zhang L. Gender schema development: a meta-analysis. Front Psychol. 2022;13:923456.

[26] Güven G Ekiz Kaya M. Sanat eğitiminde toplumsal cinsiyet algıları. Enderun. 2024;8(2):171 203. (doi: 10.59274/enderun.1544464)

[27] McMillan JH Schumacher S. Research in Education: Evidence-Based Inquiry. 7th ed. Boston (MA): Pearson; 2014.

[28] Christensen LB Johnson RB Turner LA. Research Methods, Design, and Analysis. 12th ed. Boston (MA): Pearson; 2015.

[29] Altınova HH Duyan V. Toplumsal cinsiyet algısı ölçeği geliştirme çalışması. Turk Stud. 2013;8(8):9 22.

[30] Cohen J. Statistical Power Analysis for the Behavioral Sciences. 2nd ed. Hillsdale (NJ): Lawrence Erlbaum Associates; 1988.

[31] Dalkılıç S. Yaş grupları arasında toplumsal cinsiyet algısı. Toplum Cinsiyet Araşt Derg. 2019;4(3):123 138.

[32] Kılıç A Yılmaz B Arslan C Çocuklarda toplumsal cinsiyet algısı. Çocuk Gelişim Araşt Derg. 2014;3(1):45 58.

[33] Leaper C Friedman CK. Gender and socialization. In: Damon W , Lerner RM , eds. Handbook of Child Psychology. 6th ed. New York (NY): John Wiley & Sons; 2007:561 645.

[34] McDaniel A. Women’s advantage in higher education. Demography. 2015;52(1):203 225.

[35] UNITED NATIONS EDUCATIONAL, SCIENTIFIC AND CULTURAL ORGANIZATION. Global Education Monitoring Report: Gender Report 2019. Paris (FR): UNESCO; 2019.

[36] Özaydınlık YDDK. Toplumsal cinsiyet ve eğitim. Sos Polit Çalış Derg. 2014;33:1 18.

[37] Ridgeway CL. Framed by Gender: How Gender Inequality Persists in the Modern World. Oxford (UK): Oxford University Press; 2011.

[38] Risman BJ. Where the Millennials Will Take Us: A New Generation Wrestles with the Gender Structure. Oxford (UK): Oxford University Press; 2018.

[39] Tenenbaum HR Leaper C. Are parents’ gender schemas related to their children’s gender-related cognitions? A meta-analysis. Dev Psychol. 2002;38(4):615 630. (doi: 10.1037/0012-1649.38.4.615) 12090490

[40] Williams JC Berdahl JL Vandello JA. Beyond work-life ‘integration’. Annu Rev Psychol. 2016;67:515 539. (doi: 10.1146/annurev-psych-122414-033710) 26442669

